# Artificial Intelligence in Residency Recruitment: Impact on Equity

**DOI:** 10.1212/NE9.0000000000200150

**Published:** 2024-08-30

**Authors:** Rachel Gottlieb-Smith, Kathryn Xixis, Jaclyn M. Martindale, Jessica H.R. Goldstein, Justin Rosati

**Affiliations:** From the Department of Pediatrics (R.G.-S.), University of Michigan, Ann Arbor; Department of Neurology (K.X.), University of Virginia, Charlottesville; Department of Neurology (J.M.M.), Wake Forest University School of Medicine, Winston-Salem, NC; Department of Neurology (J.H.R.G.), University of Minnesota, Minneapolis; and Department of Neurology (J.R.), University of Rochester, NY.

Artificial intelligence (AI) has the potential to revolutionize neurology education,^1^ including in residency recruitment. Machine learning (ML) is a growing branch of AI that focuses on identifying patterns in large data sets, at times allowing machines to later self-learn and adapt over time; innovators are already integrating ML into decision-support tools for residency application review.^2^ Natural language processing (NLP), another subfield of AI that seeks to understand and process human language, may be integrated within ML to enable machines to process unstructured text, such as personal statements and letters of recommendation.

As ML and NLP use expands in application review, neurology educators must understand how AI-derived tools may affect equity in the recruitment process. Programs that choose to integrate AI in resident selection should harness the potential for AI to reduce bias and mitigate unintentional biases that may result from AI adoption (Figure).

**Figure:**
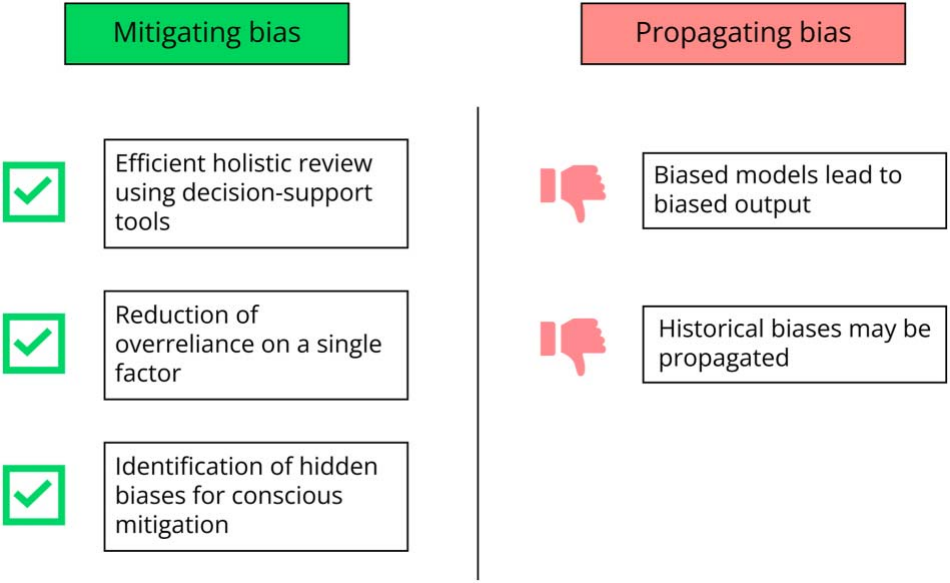
Artificial Intelligence in Residency Recruitment: Mitigating or Propagating Bias .

## Harnessing AI for Bias Reduction in Neurology Recruitment

Human application review is time-intensive, which may lead to “shortcuts” that exacerbate biases.^3^ Programs may use simplistic cutoffs such as examination scores—which may themselves be biased—to downselect applicants.^2^ For example, consider a neurology applicant who initially failed a national certification examination. Based on traditional approaches, their application may never be reviewed in depth by a neurology recruitment team, although the student shows great promise in neurology through extensive neurology-specific research experiences and excellent neurology clerkship performance. The increased efficiency afforded by AI will allow for more timely holistic review of all applications, including this student's, reducing bias based on a single parameter. Dr. Burk-Rafel and team exemplified this through a novel ML-based decision-support tool for application review.^2^ The tool was aligned with the program's reported screening factors and demonstrated inclusion of applicants who were initially inadvertently excluded by human review related to large application numbers or overreliance on a single factor. Neurology programs should develop and harness similar tools allowing for consideration of neurology-specific skills and attributes within the initial application screening process.

AI can also identify hidden biases in residency applications, such as bias in letters of recommendation revealed by NLP, allowing for explicit bias mitigation. For example, a study of general surgery residency letters of recommendation revealed gender bias, even when stratified by clerkship grades.^4^ This concern is not specific to surgery. Consider a woman neurology applicant whose letter of recommendation cites strength in communication with less focus on achievements compared with a man with overall similar clinical performance. The woman candidate may be at risk of less serious consideration by the recruitment team than her peer.^5^ By making biases in letters and assessments more explicit with use of AI tools, reviewers may be able to mitigate these biases through conscious review—or these biases may be reduced through incorporation into an AI decision-support algorithm tool.

## Mitigating Potential Bias of AI in Neurology Recruitment

Understanding limitations of AI systems is essential for appropriate use of the technology. For instance, an AI decision-support tool may inadvertently increase bias in application review if models on which the tool is based are biased. As AI use expands, it may be challenging for human “checks” to update the processes used to drive AI decision making in real time. Consider a program using a ML-based decision-support tool for application review this past season. If the algorithm is created based on past year's review, the new ERAS Impactful Experiences Section—which allows applicants to highlight resilience—may not be incorporated.^6^ While the program may highly value resilience, particularly given the high degree of burnout among neurologists and neurology trainees,^7,8^ the AI review may miss critical information in this section. A human reviewer might recognize the need to update the rubric after careful application review, but AI may not. Bias may be introduced against applicants who overcame hardships but did not have the bandwidth to participate in other activities with greater algorithmic representation. Programs need to ensure that any rubric used by AI is up-to-date, fair, and reflective of the program's mission.

Similarly, because AI is often trained on a historical data set, such as past applications or curriculum vitae from previous successful residents, historical biases may inadvertently be propagated or even magnified. The AI system may train itself to prefer applicants of specific demographics if these demographics had greater representation in previous successful resident groups. A cautionary tale from Amazon.com, Inc. of an AI resume review program exemplifies this.^9^ The system was trained by observing patterns in resumes submitted to the company over 10 years—most of which came from men, reflecting the greater male representation in the industry at the time. Because of this historical bias, the system learned to prefer male candidates and developed an antiwomen bias. Such a flaw would be particularly consequential in neurology, considering field-specific historical biases; most previous successful neurology candidates have been men from US MD-granting institutions and from ethnic groups not traditionally considered underrepresented in medicine.^10^ It is imperative that any historical data set used for residency applicants is carefully assessed for bias.

## Future Considerations

AI is here to stay. It has potential to improve residency recruitment and selection equity, with careful human oversight and awareness of potential pitfalls. As specific AI tools become available for application review, there is an urgent need to establish practice guidelines for neurology trainee recruitment teams. Programs that adopt this evolving area of technology must partake in continuous training, retraining, and evaluation of AI support tools used in the recruitment process, with attention to the effect on equity. We recommend periodic holistic review of a subset of applications for continuous model training as a countermeasure to potential model bias. Ultimately, as neurology educators, we must embrace AI, which is poised to be a powerful and useful tool in neurology residency recruitment.

## Appendix Authors

**Table:**

Name	Location	Contribution
Rachel Gottlieb-Smith, MD, MHPE	Department of Pediatrics, University of Michigan, Ann Arbor	Drafting/revision of the manuscript for content, including medical writing for content
Kathryn Xixis, MD	Department of Neurology, University of Virginia, Charlottesville	Drafting/revision of the manuscript for content, including medical writing for content
Jaclyn M. Martindale, DO	Department of Neurology, Wake Forest University School of Medicine, Winston-Salem, NC	Drafting/revision of the manuscript for content, including medical writing for content
Jessica H.R. Goldstein, MD	Department of Neurology, University of Minnesota, Minneapolis	Drafting/revision of the manuscript for content, including medical writing for content
Justin Rosati, MD	Department of Neurology, University of Rochester, NY	Drafting/revision of the manuscript for content, including medical writing for content
