# Artificial Intelligence in Residency Recruitment: Impact on Equity

**DOI:** 10.1212/NE9.0000000000200165

**Published:** 2024-11-05

In the Viewpoint article “Artificial Intelligence in Residency Recruitment: Impact on Equity” by Gottlieb-Smith et al.,^1^ the authors should be listed in the following order: Rachel Gottlieb-Smith, MD, MHPE; Kathryn Xixis, MD; Jaclyn M. Martindale, DO; Justin Rosati, MD; Jessica H.R. Goldstein, MD. The article has been replaced by a corrected version. The authors regret the error.
